# Anti‐dsDNA, anti‐nucleosome, anti‐C1q, and anti‐histone antibodies as markers of active lupus nephritis and systemic lupus erythematosus disease activity

**DOI:** 10.1002/iid3.401

**Published:** 2021-01-20

**Authors:** Xiaoying Shang, Lisheng Ren, Guirong Sun, Teng Yu, Yuan Yao, Lin Wang, Fenghai Liu, Lijun Zhang, Xiaqin He, Mingjun Liu

**Affiliations:** ^1^ Department of Clinical Laboratory The Affiliated Hospital of Qingdao University Qingdao China; ^2^ Department of Clinical Laboratory Qingdao Municipal Hospital Qingdao China

**Keywords:** autoantibodies, lupus nephritis, systemic lupus erythematosus

## Abstract

**Introduction:**

Previous studies of anti‐dsDNA, nucleosome (Nucl), histone (His), and C1q antibodies have revealed their clinical value in systemic lupus erythematosus (SLE). However, the correlation between four autoantibodies and SLE activity, lupus nephritis (LN) remains controversial, and data are insufficient on longitudinal monitoring. This study aimed at evaluating the value of these autoantibodies in active LN, and their performance on cross‐sectional evaluating and longitudinal monitoring of SLE disease activity.

**Methods:**

Serum levels of four autoantibodies in 114 SLE patients, 219 other autoimmune disease patients (OAD), and 59 healthy controls were assayed by a quantitative immunoassay. Sera of 38 inpatients were obtained again after treatment.

**Results:**

We found that serum levels of four autoantibodies were significantly higher in SLE than OAD patients (*p* < 001), active LN than non‐renal SLE patients (*p* < .05), and higher in SLE patients with moderate and severe disease activity than mild disease activity (*p* < .01). Horizontally, serum level of each autoantibody was correlated with SLE disease activity index (SLEDAI) (*p* < .05), and correlation coefficient of anti‐dsDNA was the highest (*r* = .585). For longitudinal monitoring, the decreased levels of four autoantibodies were found following treatment (*p* < .001). Serum level variations of these antibodies were positively correlated with variations of SLEDAI (*p* < .05). The correlation coefficient of anti‐Nucl was the highest (*r* = .629). Although the levels of C3 and C4 increased after treatment, the change was not related to the change of SLEDAI (*p* > .05).

**Conclusions:**

Anti‐C1q, anti‐dsDNA, anti‐Nucl, and anti‐His perform well in diagnosing active LN and monitoring SLE disease activity. They could be indicators of active LN and SLE disease activity.

## Graphical abstract

Anti‐C1q, anti‐dsDNA, anti‐Nucl, and anti‐His perform well in diagnosing active LN and monitoring SLE disease activity. They could be indicators of LN and SLE disease activity.

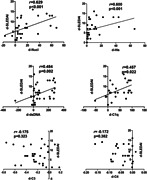

**Abbreviations**
C3: complement 3
C4: complement 4
HC: healthy controls
His: Histone
hrv: higher than reference range values
LN: lupus nephritis
lrv: lower than reference range values
Nucl: nucleosome
OR: odds ratio
ROC‐AUC: the area under the curve of receiver operating characteristic curve
SLE: systemic lupus erythematosus
SLEDAI: systemic lupus erythematosus disease activity index

## 1 INTRODUCTION

Systemic lupus erythematosus (SLE) is a systemic autoimmune disease characterized by the presence of autoantibodies particularly against components of the cell nucleus.^1^, ^2^ Kidney is one of the most commonly involved organs in SLE. Prevalence of several autoantibodies such as anti‐dsDNA, nucleosome (Nucl), histone (His), and C1q has been studied in SLE patients to evaluate their clinical significance and their value as a marker of lupus nephritis (LN).^3^, ^4^, ^5^, ^6^, ^7^, ^8^ However, their correlation with LN and SLE disease activity remains controversial. Such uncertainties deserve further clarification.

According to the Systemic Lupus Erythematosus Disease Activity Index (SLEDAI) 2000, two scores are added when the result of anti‐dsDNA is positive.^9^ Qualitative methods for detection of autoantibodies such as indirect immunofluorescence, immunoblotting, and enzyme‐linked immunosorbent assays have been available in most of clinical laboratories for many years. With the development of detection technology, high‐throughput, and quantitative detection has become a clinical need and development trend.^10^, ^11^, ^12^, ^13^ Compared with qualitative results, the quantitative ones may do better at reflecting the deterioration and improvement of disease. Furthermore, previous cross‐sectional studies demonstrated that some autoantibodies were correlated with SLEDAI, but little work has focused on the utility of autoantibodies in longitudinal monitoring of SLE disease activity.

In this study, we assayed four autoantibodies by a quantitative detection― multiplexed bead‐based flow fluorescent immunoassay in SLE patients and evaluated their value in the diagnosis of active LN, and the performance of cross‐sectional evaluating and longitudinal monitoring SLE disease activity.

## 2 MATERIALS AND METHODS

### 2.1 Patients and controls

One hundred and fourteen patients from the Department of Rheumatology (The Affiliated Hospital of Qingdao University) were diagnosed with SLE from November 2017 to April 2018. All patients fulfilled the 2012 Systemic Lupus International Collaborating Clinics (SLICC) Classification Criteria for SLE.^14^ Demographic data and laboratory findings of SLE patients were shown in Table 1. Of the 114 SLE patients, 58 patients were diagnosed with active LN according to the 2012 ACR Guidelines for Screening, Treatment and Management of Lupus Nephritis.^15^ Serum samples were collected from all SLE patients before initiating treatment. Among the 114 SLE patients, 38 individuals were inpatients. Their clinical disease activity was monitored and assessed according to the SLEDAI 2000.^9^ SLEDAI ≤6 was classified as mild disease activity, and SLEDAI >6 was classified as moderate and severe disease activity.^16^ Patients received different doses of glucocorticoid therapy appropriate to their disease activity. If necessary, immunosuppressive agents were used. Median treatment duration was 49 days (range 10–136). Their serum samples were obtained again after the treatment. Meanwhile, serum samples from 219 patients with various other autoimmune diseases (OAD) (including 181 females and 38 males, median age 52 years, range 1–86 years) and 59 healthy volunteers who were excluded autoimmune diseases were collected (including 42 females and 17 males, median age 36 years, range 5–67 years) as controls. Samples were stored at –36°C until detection. This study was conducted in accordance with the Helsinki Declaration and was approved by the Ethics Committee of the Affiliated Hospital of Qingdao University (QDFY WZ 2018‐9‐25‐03). Informed consent was obtained from each participant.

**Table 1:** Demographic data and laboratory findings of SLE patients

	*n* = 114
Proportion of females, *n* (%)	102 (89.5)
Age (years), median (range)	35 (5–84)
Lupus nephritis, *n* (%)	58 (50.9)
Non‐renal damage, *n* (%)	56 (49.1)
Anti‐dsDNA (IU/ml), median (IQR)	25.66 (6.75–95.38)
Anti‐Nucl (AI), median (IQR)	0.43 (0.17–3.34)
Anti‐C1q (U/ml), median (IQR)	4.71 (1.81–14.64)
Anti‐His (AI), median (IQR)	0.73 (0.41–2.41)
SLEDAI, scores, mean ± SD	10 ± 0.7
Newly diagnosed	37
Disease duration (years), median (range)	0.75 (0.03–8)

Abbreviations: AI, antibody index; IQR, interquartile range; SD, standard deviation; SLE, systemic lupus erythematosus.

### 2.2 The quantitative immunoassay of autoantibodies

The simultaneous determination of autoantibodies to four different antigens (dsDNA, Nucl, C1q, and His) was performed by a multiplexed bead‐based flow fluorescent immunoassay (Tellgen Co., LTD.). For anti‐C1q antibody, the cutoff value recommended by the manufacturer was more than 10 U/ml. For anti‐dsDNA antibody, the cutoff value was more than 18 IU/ml. The cutoff value of anti‐Nucl and anti‐His was more than 1.0 antibody index (AI). The operation was carried out according to the instructions of the manufacturer.

### 2.3 The detection of complement 3 (C3) and complement 4 (C4)

The serum levels of C3 and C4 were detected by immunonephelometry (Siemens BN‐Ⅱ system). The serum samples of 103 patients from 114 SLE patients were collected and tested.

### 2.4 Statistical analysis

The maximum detectability of anti‐dsDNA was 300 IU/ml, values that were greater than 300 were taken as 300. Similarly, anti‐C1q >100 U/ml were taken as 100. Continuous variables were expressed as median with interquartile range (IQR) for non‐normal distribution and mean ± SD (standard deviation) for normal distribution. Continuous variables without normal distribution were compared using Mann–Whitney *U* test (serum levels of antibodies in active LN vs. non‐renal SLE, active LN vs. healthy controls (HC), all SLE vs. HC, active LN vs. OAD, all SLE vs. OAD, mild vs. moderate and severe). *χ* ^2^ test was used to compare ratios between active LN and non‐renal SLE, mild and moderate/severe. For comparisons of antibodies before and after treatment, Wilcoxon matched‐samples signed rank‐sum test was used for non‐normally distributed difference between the pairs, and paired *t* test was used for normally distributed difference between the pairs (only C3). Correlations between the serum level of each antibody or C3, C4, and SLEDAI were studied with Spearman's rank correlation. Correlations between the serum level variation of each antibody and variation of SLEDAI were studied with Spearman's rank correlation, and with Pearson correlation for C3. Statistical significance was set at *p* < .05. SPSS 24.0 and GraphPad Prism 5 were used for data storage and analysis.

## 3 RESULTS

### 3.1 Diagnostic utility of autoantibodies in SLE

**Figure 1:**
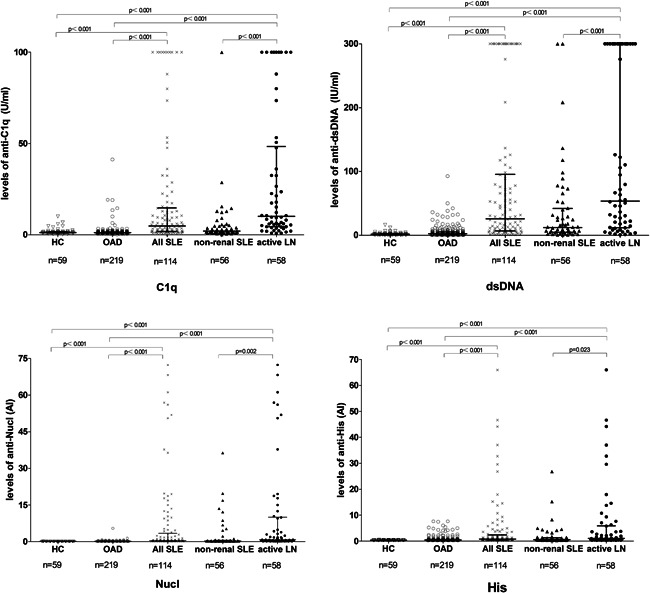
Comparison of serum levels of four autoantibodies in all SLE patients, active LN patients, non‐renal SLE patients, other autoimmune disease (OAD) patients, and healthy controls (HC). Data were presented as median with interquartile range. Serum levels were compared using Mann–Whitney *U* test. LN, lupus nephritis; SLE, systemic lupus erythematosus

The serum levels of anti‐C1q, anti‐dsDNA, anti‐Nucl, and anti‐His were significantly higher in SLE patients than OAD patients (*p* < .001) and healthy controls (*p* < .001) (Figure 1). The medians with IQR of anti‐C1q in all SLE patients, OAD, and healthy controls were 4.71 (1.81–14.64), 1.13 (0.84–1.57), and 1.14 (0.86–1.71)  U/ml, respectively. The medians with IQR of anti‐dsDNA in three groups above were 25.66 (6.75–95.38), 2.24 (1.23–5.23), and 1.50 (0.53–2.57) IU/ml, respectively. The medians with IQR of anti‐Nucl in three groups above were 0.43 (0.17–3.34), 0.14 (0.12–0.19), and 0.12 (0.11–0.13) AI, respectively. The medians with IQR of anti‐His in three groups above were 0.73 (0.41–2.41), 0.39 (0.31–0.59), and 0.33 (0.27–0.35) AI, respectively.

**Figure 2:**
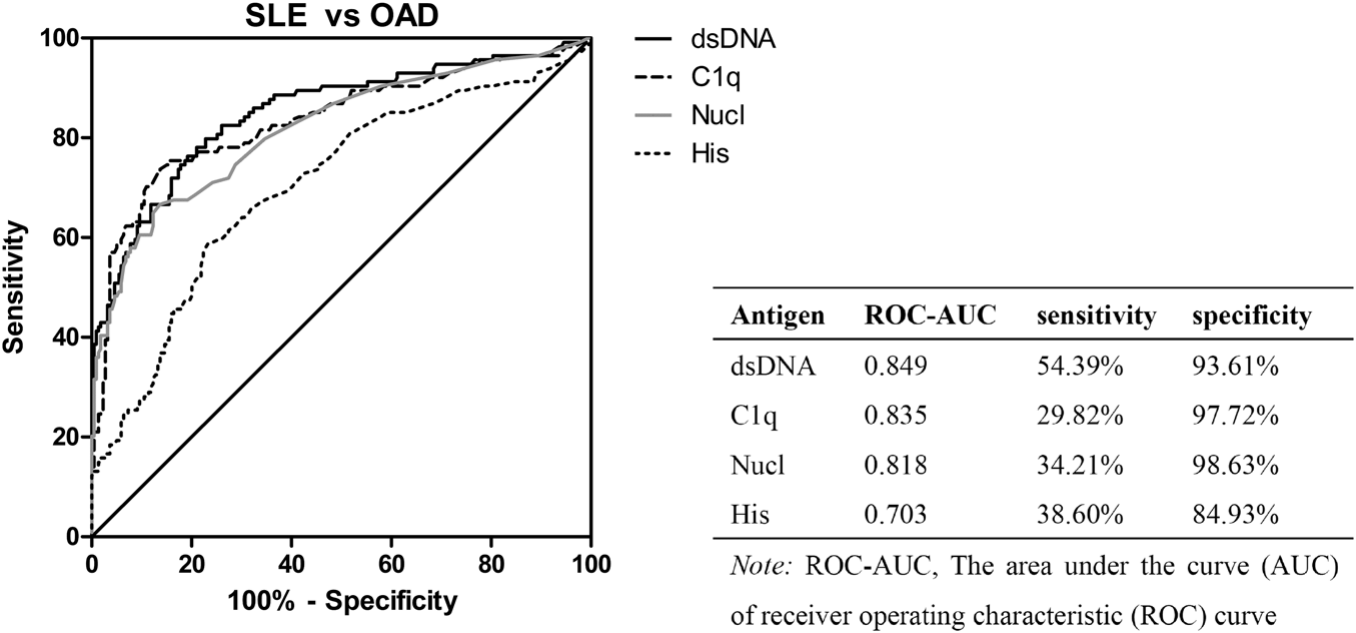
ROC curves reflect the performance of four antibodies on discriminating between SLE and other autoimmune disease (OAD) patients. The sensitivity and specificity of diagnosing SLE (OAD as control) were in the table. ROC, receiver operating characteristic  curve; SLE, systemic lupus erythematosus

The greatest area under the curve (AUC) of the receiver operating characteristic (ROC) curve was 0.849 (anti‐dsDNA). The specificity of anti‐Nucl was the highest (98.63%) (Figure 2).

### 3.2 Diagnostic utility of autoantibodies in active LN

The serum levels of anti‐C1q, anti‐dsDNA, anti‐Nucl, and anti‐His were significantly higher in active LN patients than non‐renal SLE patients (*p* < .05), in active LN patients than healthy controls or OAD (*p* < .001) (Figure 1). The medians with IQR of anti‐C1q in active LN patients and non‐renal SLE patients were 10.04 (4.39–48.37) and 1.99 (1.21–4.73) U/ml. The medians with IQR of anti‐dsDNA in two groups above were 53.38 (11.79–300.00) and 12.18 (4.67–41.87) IU/ml. The medians with IQR of anti‐Nucl in two groups above were 0.88 (0.24–9.99) and 0.27 (0.17–0.59) AI. The medians with IQR of anti‐His in two groups above were 0.99 (0.44–5.88) and 0.59 (0.40–1.30) AI.

**Figure 3:**
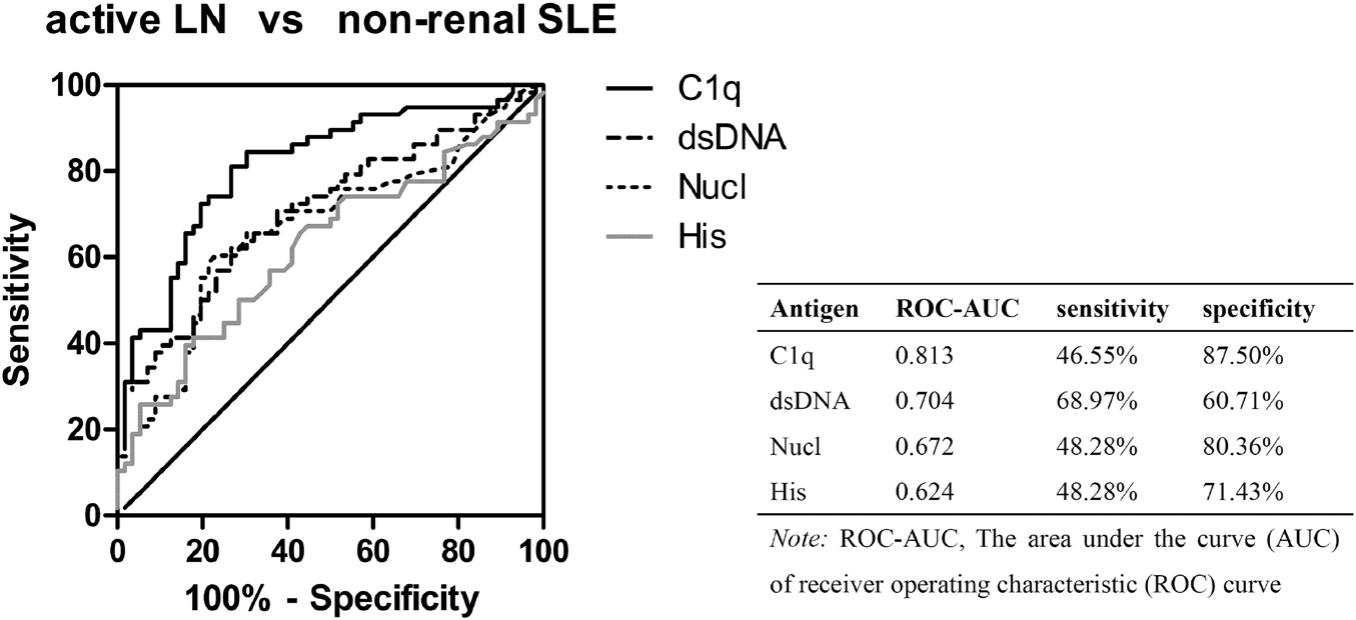
ROC curves reflect the performance of four antibodies on discriminating between active LN and non‐renal SLE patients. The sensitivity and specificity of diagnosing active LN were in the table. LN, lupus nephritis; SLE, systemic lupus erythematosus

The ROC‐AUC of anti‐C1q was the greatest (0.813) in differentiating active LN from non‐renal SLE patients. The sensitivity of anti‐dsDNA was the highest (68.97%). The specificity of anti‐C1q was the highest (87.50%) (Figure 3). The percentage of the four antibodies higher than reference range values (4‐hrv) was 27.59% in active LN patients and 7.14% in non‐renal SLE patients (*p* = .009, odds ratio [OR] = 4.95). The percentage of the four antibodies lower than reference range values (4‐lrv) was 22.41% in active LN patients and 50.00% in non‐renal SLE patients (*p* = .002, OR = 0.29) (Table 2).

**Table 2:** The combined detection results of four antibodies in differentiating active LN from non‐renal SLE patients

Antigen	Active LN (*n* = 58; %)	Non‐renal (*n* = 56; %)	OR (95% CI)	*p* Value
hrv: C1q, dsDNA, Nucl, His	16 (27.59%)	4 (7.14%)	4.95 (1.54–15.94)	.009
lrv: C1q, dsDNA, Nucl, His	13 (22.41%)	28 (50.00%)	0.29 (0.13–0.65)	.002

Abbreviations: CI, confidence interval; hrv, higher than reference range values, lrv, lower than reference range values; OR, odds ratio; SLE, systemic lupus erythematosus.

### 3.3 Utility of autoantibodies in differentiating moderate and severe from mild SLE disease activity

**Figure 4:**
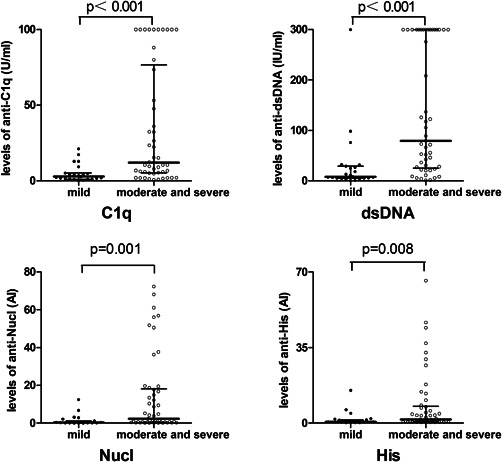
Comparison of serum levels of four autoantibodies in mild disease activity patients with moderate and severe disease activity patients. Data were presented as median with interquartile range. Serum levels were compared using Mann–Whitney *U* test

The serum levels of anti‐C1q, anti‐dsDNA, anti‐Nucl, and anti‐His were significantly higher in SLE patients with moderate and severe disease activity than mild disease activity (*p* < .01) (Figure 4). The medians with IQR of anti‐C1q, anti‐dsDNA, anti‐Nucl, and anti‐His in SLE patients with moderate and severe disease activity were 12.15 (5.25–76.74) U/ml, 79.48 (25.43–300.00) IU/ml, 2.38 (0.41–18.23) AI, and 1.74 (0.65–7.93) AI, respectively. The medians with IQR of anti‐C1q, anti‐dsDNA, anti‐Nucl, and anti‐His in patients with mild disease activity were 2.86 (1.12–5.14) U/ml, 8.21 (5.02–29.13) IU/ml, 0.39 (0.16–1.15) AI, and 0.71 (0.38–1.53) AI, respectively.

**Figure 5:**
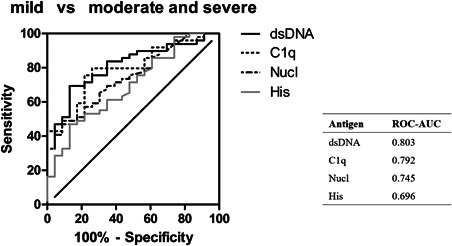
ROC curves reflect the performance of four antibodies on discriminating moderate and severe disease activity from mild disease activity patients. AUC, area under the curve; ROC, receiver operating characteristic curve

The ROC‐AUC of anti‐dsDNA was the largest (0.803) in differentiating moderate and severe from mild disease activity (Figure 5). The percentage of the four antibodies higher than reference range values (4‐hrv) was 36.73% in patients with moderate and severe disease activity and 4.35% in mild activity patients (*p* = .009, OR = 12.77). The percentage of the four antibodies lower than reference range values (4‐lrv) was 14.29% in patients with moderate and severe disease activity and 39.13% in mild activity patients (*p* = .018, OR = 0.26) (Table 3).

**Table 3:** The combined detection results of four antibodies in differentiating moderate and severe from mild disease activity

Antigen	Moderate and severe (*n* = 49; 68.06%)	Mild (*n* = 23; 31.94%)	OR (95% CI)	*p* value
hrv: C1q, dsDNA, Nucl, His	18 (36.73%)	1 (4.35%)	12.77 (1.58‐102.89)	.009
lrv: C1q, dsDNA, Nucl, His	7 (14.29%)	9 (39.13%)	0.26 (0.08‐0.83)	.018

Abbreviations: CI, confidence interval; hrv, higher than reference range values; lrv, lower than reference range values; OR, odds ratio.

### 3.4 The correlation between SLEDAI and autoantibodies, C3, C4

**Figure 6:**
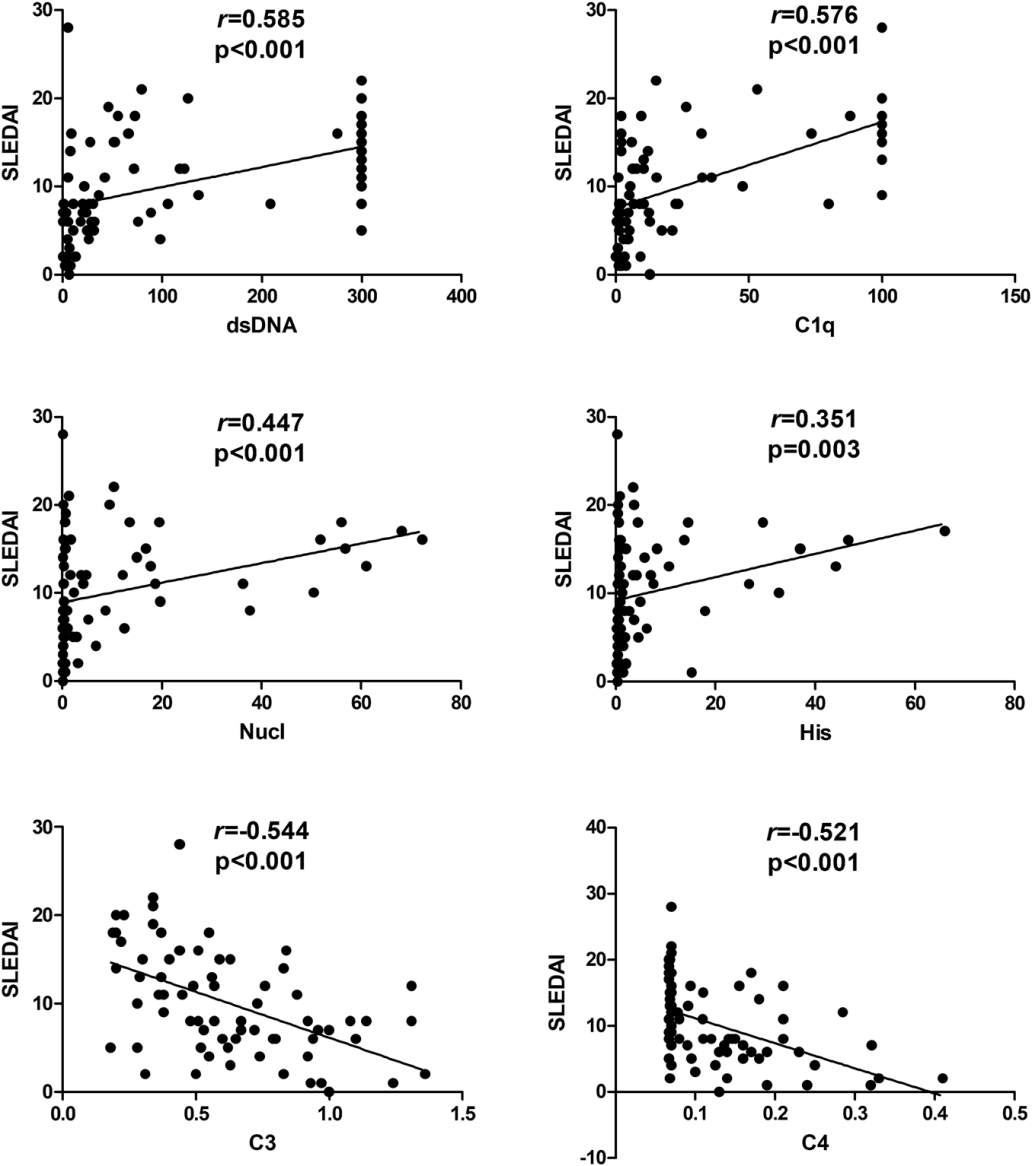
Correlations between the serum levels of four antibodies, C3, C4, and SLEDAI scores in 72 SLE patients. *r*, correlation coefficient; Spearman's rank correlation were used. C3, complement 3; C4, complement 4; SLE, systemic lupus erythematosus; SLEDAI, systemic lupus erythematosus disease activity index

The disease activity of 72 SLE patients was assessed according to SLEDAI 2000. The serum level of each autoantibody was positively correlated with SLEDAI (*p* < .05). The correlation coefficient of anti‐dsDNA was the highest (*r* = .585). The medians with IQR of C3 and C4 were 0.630 (0.433–0.920) g/L and 0.120 (0.07–0.183) g/L, respectively. The levels of C3 and C4 were negatively related to SLEDAI (*p* < .001) (Figure 6).

### 3.5 The decline in serum levels of autoantibodies after treatment

**Figure 7:**
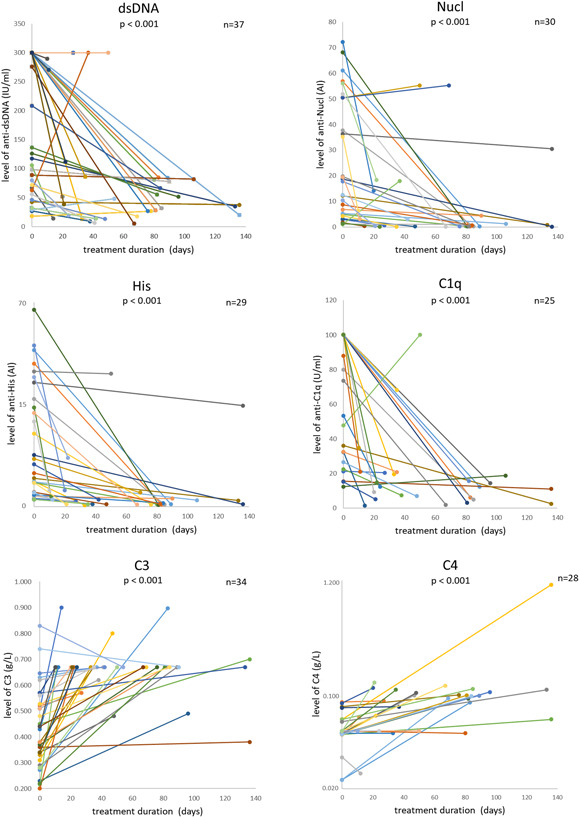
The serum levels of four antibodies, C3, and C4 in some of SLE patients were tested before and after treatment. Every straight line represented a patient. The abscissa was the time interval between two tests (treatment duration). *n*, the numbers of cases with antibody level higher/complements level lower than the reference range values before treatment. Wilcoxon matched‐samples signed rank‐sum test was used, and paired *t* test was used (only C3). C3, complement 3; C4, complement 4; SLE, systemic lupus erythematosus

The change in serum levels of autoantibodies was assessed in 38 inpatients after treatment. We selected cases with antibody levels higher than the reference range values and cases with complements level lower than the reference range values before treatment, and the numbers (*n*) of cases were marked in Figure 7. The abscissa was the time interval between two tests (treatment duration). The decreased serum levels of anti‐dsDNA, anti‐Nucl, anti‐His, and anti‐C1q were found after therapies (*p* <. 001) (Figure 7).

**Figure 8:**
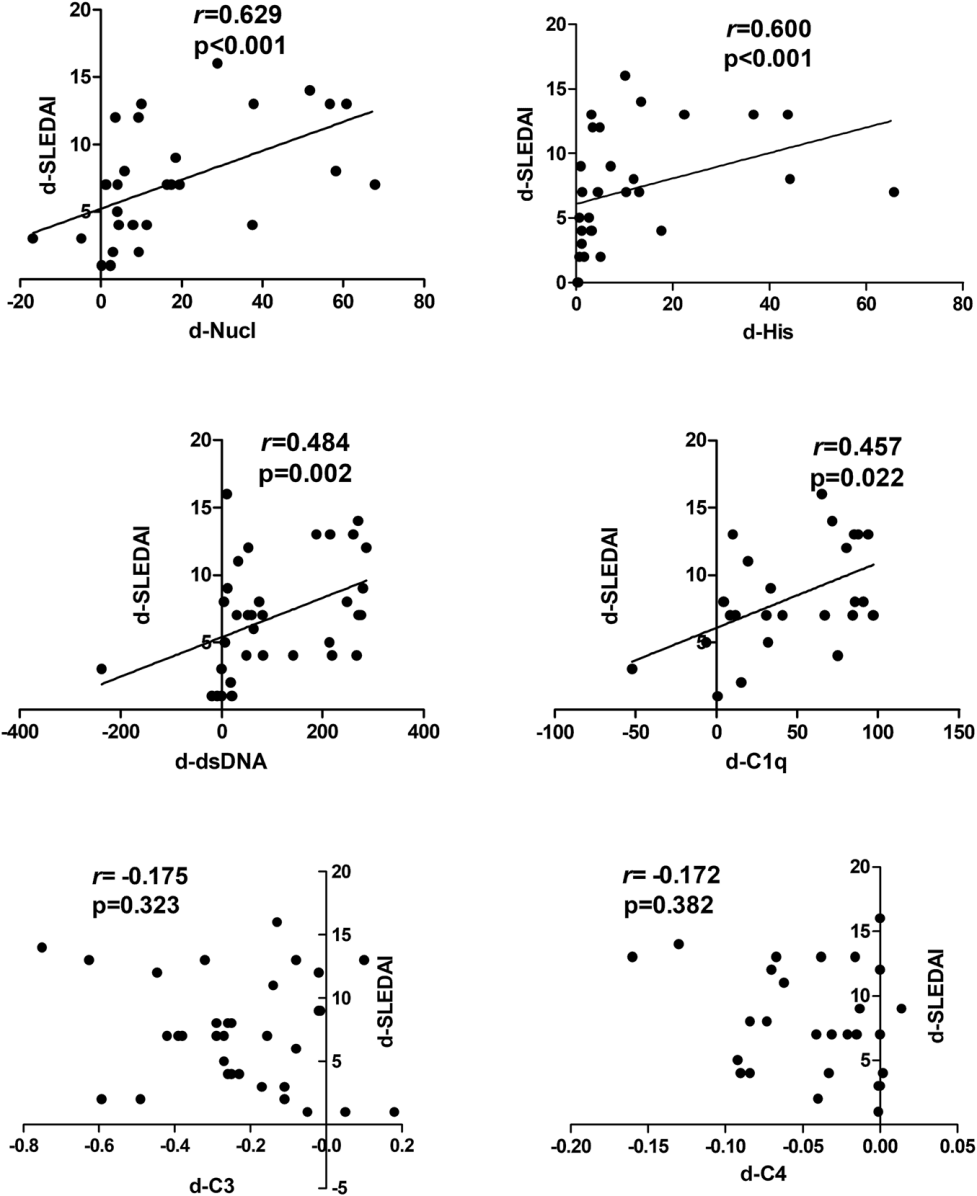
Correlations between the serum level variation of each antibody and variation of SLEDAI. The correlation coefficient (*r*) was illustrated in the graph. d‐SLEDAI, the difference of SLEDAI = SLEDAI before treatment−SLEDAI after treatment. d‐dsDNA, the difference of the serum level of anti‐dsDNA = the serum level of anti‐dsDNA before treatment−the serum level of anti‐dsDNA after treatment, the calculations of other antibodies and C3, C4 were analogous. Spearman's rank correlation and Pearson correlation (only for C3) were used. C3, complement 3; C4, complement 4; SLEDAI, systemic lupus erythematosus disease activity index

We evaluated the correlation between the variation in serum levels of these autoantibodies and the variation in SLEDAI. At the same time, the correlation between the change in serum levels of C3, C4, and the change in SLEDAI was evaluated. The change in serum level of each autoantibody was positively correlated with change in SLEDAI (*p* < .05). The correlation coefficient of anti‐Nucl was the highest (*r* = .629). However, the changes in the levels of C3 and C4 were not related to the change in SLEDAI (*p* > .05) (Figure 8). Notably, the levels of C3 and C4 were negatively related to SLEDAI horizontally and decreased after treatment as stated above.

## 4 DISCUSSION

The clinical performance and course of SLE is heterogeneous, which hinders early diagnosis and the monitoring of disease progression. Although clinical evaluation is the foundation of SLE patient management, it is limited. Additional means to confirm diagnosis and define disease activity are required. The detection of autoantibodies plays an important role in the diagnosis of SLE and LN. They may provide guidance on the response to specific therapies for patients. The level of anti‐dsDNA antibody acts as a diagnostic marker. Fluctuations in anti‐dsDNA and complement can be used to monitor disease activity.^9^ Although these traditional biomarkers are widely used now, they are insufficient in certain clinical situations, such as the reliable identification of active patients and longitudinal monitoring of disease activity.

Our previous study showed that multiplexed bead‐based flow fluorescent immunoassay was in good agreement with traditional immunoblot when testing specific autoantibodies.^17^ In the present study, we quantitatively detect autoantibodies in SLE patients, and assessed their diagnostic value in active LN and performance as disease activity and longitudinal monitoring biomarkers.

The kidney is one of the major involved organs in SLE. The identification of serum biomarkers for predicting LN and reflecting renal activity is significant.^3^ In our study, anti‐C1q, anti‐dsDNA, anti‐Nucl, and anti‐His showed good utility in the diagnosis of SLE renal involvement. Anti‐C1q could be the best diagnostic marker for LN. The percentage of the 4‐hrv or 4‐lrv was different in active LN and non‐renal SLE, which showed that 4‐hrv or 4‐lrv could diagnose LN. The OR of the patient group with 4‐hrv was 4.95, which showed the tendency to active LN occurrence was 4.95 times higher than the tendency in non 4‐hrv. The OR of the patient group with 4‐lrv was 0.29 (1/3.45), which showed the tendency to active LN occurrence was 1 in 3.45 of that in non 4‐lrv. A possible correlation of these antibodies with SLE renal damage has been proven previously.^4^, ^6^, ^7^ However, Katsumata et al.^18^ put forward that anti‐C1q did not correlate with LN. In contrast, a previous study suggested that anti‐C1q performed relatively good sensitivity and specificity in the diagnosis of LN.^19^ This difference in results may be due to the differences in demographic characteristics of subjects and different antigen preparations of antibodies various manufactures. Some study showed that anti‐C1q may also directly bind to C1q bound to the glomerular immune complexes and thus increase the damage.^20^ The presence of anti‐dsDNA antibodies was correlated with certain histopathological indices reflecting active renal disease.^7^ In previous research, anti‐DNA antibodies (fluctuated in levels with active nephritis) were enriched in renal eluates, and deposited in the kidney when injected into normal mice, all of which has proved the important role of anti‐DNA in immune‐complex‐mediated renal pathology.^21^ Previous research showed active LN was associated with an increased level of anti‐Nucl.^22^

Each of the four antibodies could differentiate moderate and severe disease activity from mild disease activity. The ROC‐AUC of anti‐dsDNA was the largest, whose diagnostic utility of moderate and severe disease activity was the best. The prevalence for 4‐hrv or 4‐lrv in patients with moderate and severe disease activity was significantly different from patients with mild disease activity, which suggested that 4‐hrv or 4‐lrv could also distinguish moderate and severe from mild disease activity. The OR of the patients with 4‐hrv was 12.77, which shows the possibility to moderate and severe disease activity was 12.77 times higher than the possibility in non 4‐hrv patients. The OR of the patients with 4‐lrv was 0.26 (1/3.86), which suggested the tendency to mild disease activity was 3.86 times higher than the tendency in non 4‐lrv patients.

Although the available biomarkers of anti‐dsDNA^20^, ^22^, ^23^ and other autoantibodies are useful for longitudinal monitoring of SLE disease activity, research which has quantitatively detected specific autoantibodies by flow immunofluorescence assay and evaluated their value of longitudinal monitoring of SLE disease activity are few. Horizontally, the levels of anti‐dsDNA was correlated to SLEDAI the best in the present study. For longitudinal monitoring SLE disease activity, the disease activity has dropped after treatment in most of the subjects. It showed that the levels of these autoantibodies could reflect the response to the treatment. We found that the serum levels of four antibodies (anti‐dsDNA, anti‐Nucl, anti‐His, and anti‐C1q) reduced after treatment. The change in serum level of each autoantibody was positively correlated with change in SLEDAI. Notably, anti‐Nucl was the most relevant. Therefore, anti‐dsDNA performed best in evaluating disease activity horizontally, and anti‐Nucl was the most sensitive antibody for longitudinal monitoring SLE disease activity and therapeutic efficacy. The possible reasons for the correlation between anti‐Nucl and disease activity are: in pathological state of SLE, a large number of apoptotic cells produces excessive nucleosomes, which induces cell necrosis and inflammatory reaction.^24^ At the same time, nucleosomes could stimulate the production of anti‐Nucl, which induces immune abnormalities.^25^ The reasons for the correlation between the elevated level of anti‐C1q antibody and SLE disease activity may be as follows: complements are activated in SLE patients. C1q, which is the first component of the complement system and activator of the classical pathway, is produced in large quantities and binds to apoptotic cells, exposing the epitope to the immune system.^26^ The immune system is stimulated to produce anti‐C1q antibody and form immune complexes containing C1q and anti‐C1q autoantibodies which will result in full activation of the classical pathway of the complement system, leading to tissue injury.^27^ Moreover, anti‐C1q antibody can hinder the clearance of apoptotic cells by C1q. Defective clearance of apoptotic cells, which may become antigen, is an important hypothesis about the pathogenesis of SLE.

In our study, the level of anti‐dsDNA declined, but still remained higher than the reference range value. The criterion of anti‐dsDNA is positive or negative in SLEDAI, the score of anti‐dsDNA will not decrease, which cannot reflect the change in condition. Quantitative assay of anti‐dsDNA could be more sensitive to monitor the progression and improvement of disease. Previous data showed the utility of anti‐Nucl,^28^, ^29^, ^30^, ^31^ anti‐His,^30^, ^32^ anti‐dsDNA,^20^, ^22^, ^23^ anti‐C1q^33^, ^34^, ^35^ for monitoring SLE disease activity, which were consistent with our findings.

The levels of C3 and C4 were correlated to SLEDAI horizontally, and decreased after treatment, but the change of complements were not correlated with the change of SLEDAI. It proved that C3 and C4 could reflect the SLE disease activity, but were not sensitive enough to monitor the therapeutic efficacy. The reasons may be as follows: the individual metabolic rate of complements was heterogeneous, which may not synchronize with the changes of the disease. Some patients have been relieved, but their C3 and C4 levels have not been recovered.^36^ However, the change in serum level of each autoantibody was positively correlated with change in SLEDAI. Thus, anti‐Nucl, anti‐His, anti‐dsDNA, and anti‐C1q could be more useful than complements for longitudinal monitoring of SLE disease activity. This will provide a new perspective for clinicians to judge the therapeutic efficacy in SLE patients.

Unfortunately, considering the small number of patients for longitudinal monitoring of SLE disease activity, we acknowledge a limitation of this part of the study. Further studies with large cohorts of SLE patients are necessary to carefully evaluate the clinical utility of the quantitative immunoassay for autoantibodies. Despite its limitation, this study can clearly indicate their good utility in monitoring SLE disease activity.

In conclusion, anti‐C1q, anti‐dsDNA, anti‐Nucl, and anti‐His perform well in diagnosing active LN and in differentiating moderate and severe SLE disease activity from mild disease activity. Anti‐C1q could be the best diagnostic marker for active LN. Anti‐dsDNA performs best in differentiating moderate and severe from mild disease activity. The four autoantibodies could be more useful than C3 or C4 for longitudinal monitoring of SLE disease activity. Anti‐dsDNA performs best in evaluating disease activity horizontally, and anti‐Nucl is the most sensitive antibody for longitudinal monitoring SLE disease activity and therapeutic efficacy.

## CONFLICT OF INTERESTS

The authors declare that there are no conflict of interests.

## AUTHOR CONTRIBUTIONS

All the authors participated in the acquisition, analysis, interpretation of data or designed the study. All authors approved the final version to be published. *Wrote the paper*: Xiaoying Shang. *Played an important role in conceived, designed and supervised the study*: Mingjun Liu.

## Data Availability

The data that support the findings of this study are available from the corresponding author upon reasonable request.
